# Factors Related to Ikigai among Home-visit Rehabilitation Users Aged 75 Years and Older Receiving Home Medical and Nursing Care in Japan

**DOI:** 10.1298/ptr.E10289

**Published:** 2024-11-14

**Authors:** Eisuke KOGURE, Takeshi OHNUMA, Yuta SUGITA, Tsuyoshi HARA

**Affiliations:** 1 Acinara Home Visit Nurse Station, GOJO Incorporated, Japan; 2 Rehabilitation Progress Center Incorporated, Japan; 3 Nishinasuno General Home Care Center, Japan; 4 Department of Physical Therapy, School of Health Science, International University of Health and Welfare, Japan

**Keywords:** Instrumental activities of daily living, Cross-sectional, Index, Multi-center

## Abstract

Objective: This study investigated the factors that influence Ikigai among people aged 75 years and older using home medical and nursing care with home-visit rehabilitation. Methods: This cross-sectional study involved 66 home-visit rehabilitation users aged 75 years or older who received home medical and nursing care at 2 home-care nursing stations. The following attributes were evaluated: Ikigai-9, life-space assessment (LSA), home-based LSA (Hb-LSA), Frenchay activities index (FAI), functional independence measure (FIM), self-efficacy for activities of daily living (SEADL), self-efficacy scale for going out among community-dwelling elderly (SEGE), and physical function. The correlation between Ikigai-9 scores and each assessment was examined. Multiple regression analysis was performed using the Ikigai-9 as the dependent variable and the correlated items as independent variables. Results: The Hb-LSA, FAI, FIM, SEADL, and SEGE were correlated with Ikigai-9. Among the correlated items, the FAI was selected for multiple regression analysis. Conclusion: Home-visit rehabilitation users aged 75 and over may be more likely to feel Ikigai if they have higher instrumental ADLs.

## Introduction

In Japan, owing to the aging of the baby-boom generation, the number of people aged 75 years and older is expected to increase after 2025^1)^. Compared to persons under 75 years old, the physical function and quality of life (QOL) in elderly over 75 years old are more likely to decline after surgery^2)^. In addition to the variety of diseases they suffer^3)^, many elders over 75 years old require home care and medical services.

In these individuals, physical and mental functions are impaired owing to frailty, resulting in a decline in the QOL, instrumental activities of daily living (IADL), and activities of daily living (ADL)^4^^,^^5)^. In addition, mental function affects QOL in older adults to a greater extent than physical function^6)^. Thus, Ikigai has become a focus of research as a factor related to QOL^7)^.

Ikigai is a psychological concept broadly defined as “what makes life worth living^8)^.” Ikigai includes aspects of eudaimonic well-being (i.e., well-being that seeks important meaning and value over life, such as the purpose of life)^9^^,^^10)^, as well as aspects of hedonic well-being (i.e., well-being related to motivation, such as satisfaction from hobbies, travel, and food)^11^^–^^15)^, which has been associated with physical activity, mortality, disease risk reduction, and QOL^9^^,^^10^^,^^16^^,^^17)^. The concept of Ikigai, with its various related aspects, has also attracted attention in other countries^18^^,^^19)^.

Elderly people whose life functions, such as ADL and IADL, have declined require rehabilitation aiming to improve QOL, including Ikigai^20^^,^^21)^. For older people with physical and mental impairments, discovering Ikigai using the remaining functions is an essential rehabilitative role. Reports indicate that a comprehensive evaluation of measures, such as ADL and IADL, along with the implementation of rehabilitation, can improve Ikigai^22)^. Based on these findings, Ikigai is a broad concept that includes physical factors, such as physical function, mental factors, such as mental function, and social factors, such as hobbies and social activities. Thus, Ikigai is considered a factor affecting QOL. However, the factors related to influential Ikigai and the effects of home-visit rehabilitation in people aged 75 years or older who use long-term care insurance have not yet been clarified. In healthy older adults, Ikigai is lost with age and is related to IADL^23)^. Even in healthy elderly people, the decline in physical and mental functions, as well as social factors, is believed to negatively affect Ikigai^24)^.

Therefore, individuals older than 75 years with long-term care insurance may have lower Ikigai scores than healthy older adults. Thus, investigating the Ikigai-related functioning of people aged 75 years or older who require home medical and nursing care may contribute to the improvement of their QOL. In addition, it may help provide home-visit rehabilitation to these individuals. This study aimed to investigate the factors associated with Ikigai in elderly persons aged 75 years and older, who require home medical and nursing care, as well as home-visit rehabilitation.

## Methods

### Ethics approval and informed consent

This study was approved by the Ethics Committee of the International University of Health and Welfare (approval no. 19-Io-237). The study was conducted in accordance with the principles of the Declaration of Helsinki. Measurements were obtained after verbal and written explanations were provided to the participants or their caregivers and consent was obtained.

### Study participants

The study participants included home-visit rehabilitation users registered at the Itabashi Rehabilitation Home Visit Nursing Station and Nishinasuno General Home Care Center from August to October 2020.

Exclusion criteria were those younger than 75 years; those who declined to participate; those who found it challenging to participate in the study because of rapport formation or mental instability; those who had difficulty understanding verbal instructions or speaking owing to dementia, ventilator use, stroke, or other illness; those with difficulty in measuring owing to worsening medical conditions; and patients with incomplete data.

### Study design

This cross-sectional study involved participants from 2 home-care nursing stations. Physical or occupational therapists conducted the Ikigai and functional assessments. Basic attributes such as age, sex, duration of home-visit rehabilitation, and primary illness were collected from medical records or interviews. A physician involved in home healthcare managed the home healthcare for all participants. A home-visit rehabilitation session was conducted once or twice a week for 40–60 minutes and included basic movement practice, walking practice, and ADL/IADL practice.

### Evaluation of Ikigai

Ikigai was evaluated using the Ikigai-9^7)^. The Ikigai-9 is an evaluation method that has been proven valid and reliable in previous studies^7)^. This measure consists of 9 items: I often feel that I am happy; I want to learn something new or start; I think that I am useful for something else or society; I am relaxed mentally; I am interested in various things; I think that my existence is necessary for something or someone else; My life is abundant and fulfilling; I want to extend my possibilities; and I think that I am influencing someone. For each of the 9 items in the Ikigai-9, the purpose in life of the participants was evaluated on 5 levels: 1 = *strongly disagree*, 2 = *disagree a little*, 3 = *neither agree nor disagree*, 4 = *agree a little*, and 5 = *strongly agree*. The total score ranges from 9 (lowest) to 45 (highest). A higher total score indicated a better life purpose for the participant.

### Factors influencing Ikigai

For the factors potentially influencing Ikigai-9, we investigated physical activity, Frenchay activities index (FAI), functional independence measure (FIM), self-efficacy for activities of daily living (SEADL), self-efficacy scale for going out among community-dwelling elderly (SEGE), and physical function, such as hand grip strength and 30-second chair-stand test (CS-30), based on the International Classification of Functioning, Disability and Health.

The life-space assessment (LSA)^25)^ and home-based LSA (Hb-LSA)^26)^ were used to assess physical activity. LSA is an assessment method that calculates a score based on the range of activity, frequency of activity, and degree of independence over the past month. The scores range from 0 to 120 points, with a higher score indicating a larger living space. Hb-LSA is an assessment method that calculates the score based on the physical activity in an indoor living space during the past month, with a range of 0–120 points.

IADL was assessed using the FAI^27^^,^^28)^. The FAI is a 4-item rating system based on the frequency of applied and social activities over the past 3 or 6 months. The FAI consists of 15 items, with a score range of 0–45 points. A higher score indicates more frequent activity.

ADL was calculated using the motor and cognitive items, which are sub-items of the FIM^29)^, and the total score was obtained by summing these items. The total score consists of 18 items: 13 motor items and 5 cognitive items. Each sub-item was rated on a scale of 1–7 points, with higher scores indicating greater independence in ADL.

Self-efficacy was assessed using the SEADL^30)^ and SEGE^31)^. The SEADL assesses the degree of self-confidence in 6 ADLs and IADLs (bathing, walking around the house, answering the phone immediately, putting on and taking off clothes, simple cleaning, and shopping at a neighborhood store). The SEGE assesses the level of self-confidence using 6 items related to going out: I can go out even if my family and friends stop me; I can go out if I want to go out; I can go out even when I am reluctant; I can go out even if I pass through unpaved areas (including slippery places); I can go out without any particular purpose; I can go out for work or care for people; and I can handle going out even if I feel sick when I go out. Both the SEADL and SEGE assessed the degree of self-confidence on a 4-point scale. Scores range from 6 to 24, with higher scores indicating greater self-efficacy.

Upper limb strength was measured using grip strength, and lower limb strength was measured using the CS-30^32)^. Grip strength was measured twice on both the right and left sides, and the maximum value was used. The starting posture of the CS-30 was a chair-sitting position with the back of the participant away from the backrest and the arms crossed. The participants were instructed to stand and sit as many times as possible for 30 s, and the number of times they stood up was recorded.

### Statistical analyses

For statistical processing, the χ^2^ test and unpaired t-test were used to compare basic attributes of participants and incomplete data groups. Spearman’s rank correlation coefficient was used to correlate Ikigai-9 scores with age and each functional assessment. Multiple regression analysis was performed with the Ikigai-9 score as the dependent variable and items that indicated correlations as the independent variables. As in the previous study, the ordinal scale was converted into a dummy variable to perform the statistics^33)^.

Additionally, we included sex as an adjustment variable, which affects changes in the Ikigai and IADL^34^^,^^35)^. Independent variables were checked for multicollinearity.

For multicollinearity, independent variables with an absolute correlation coefficient of 0.9 or higher between independent variables, and those with a variance inflation factor of 10 or higher, were excluded. When multicollinearity was observed, the independent variable with a high correlation coefficient with the Ikigai-9 was selected.

IBM SPSS Statistics version 27 (IBM, Armonk, NY, USA) was used for statistical processing, and the significance level was set at 5%.

## Results

**Fig. 1.**
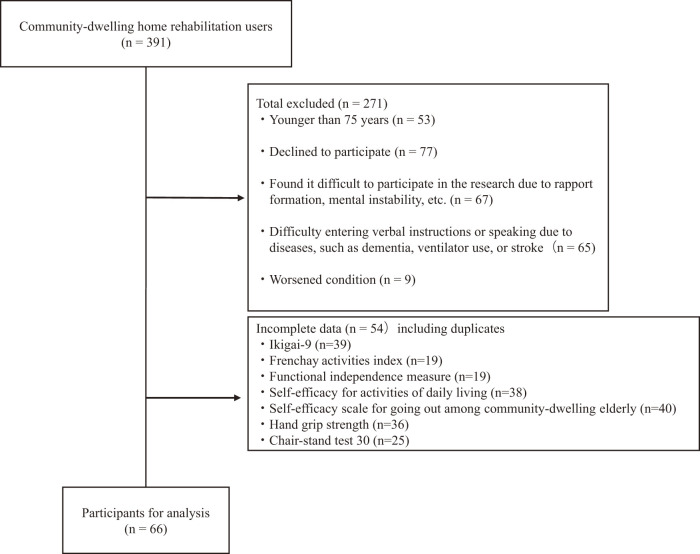
Details of the participant criteria

Of the 391 participants, 66 were included in the final analysis (Fig. 1). The attributes of the 2 groups and measurements of participants are presented in Table 1. The mean Ikigai-9 was 25.0, and the median score was 24.0. Approximately half of the participants scored lower than 25. There were no significant differences in basic attributes between the 2 groups.

**Table 1.** Attributes of participants and incomplete data group

	Participants (n = 66)		Incomplete data group (n = 54)		
	Mean ± SD n (%)	Median (25th–75th percentiles)	Mean ± SD n (%)	Median (25th–75th percentiles)	*P* value
Age (years)	83.5 ± 5.3	83.0 (79.0–86.8)	82.0 ± 7.0	81.0 (76.3–88.0)	n.s
Sex (n)					
Male	35 (53.0%)			29 (53.7%)	n.s
Female	31 (47.0%)			25 (46.3%)	
Duration of home-visit rehabilitation (months)	32.3 ± 32.8		42.1 ± 40.8		n.s
Main disease (n)					
Cerebrovascular disease	13 (19.7%)		16 (29.6%)		n.s
Musculoskeletal disease	23 (34.8%)		10 (18.5%)		
Neuromuscular disease	12 (18.2%)		16 (29.6%)		
Cardiovascular disease	4 (6.1%)		1 (1.9%)		
Respiratory disease	6 (9.1%)		1 (1.9%)		
Cancer	1 (1.5%)		1 (1.9%)		
Others	7 (10.6%)		9 (16.7%)		
Ikigai-9 (points)					
Total	25.0 ± 7.6	24.0 (20.3–30.0)			
Male	22.8 ± 7.9	23.0 (18.0–28.5)			
Female	27.4 ± 6.4	27.0 (22.5–31.0)			
LSA (points)	29.5 ± 17.1	27.0 (17.3–37.8)			
Hb-LSA (points)	66.9 ±20.3	68.0 (53.3–81.0)			
FAI (points)	7.7 ± 8.0	5.0 (1.0–12.0)			
Motor FIM (points)	72.3 ± 12.9	76.0 (65.5–79.8)			
Cognitive FIM (points)	32.5 ± 3.3	34.0 (31.0–35.0)			
Total FIM (points)	104.8 ± 14.2	108.0 (97.3–114.0)			
SEADL	13.1 ± 4.5	12.0 (10.0–16.8)			
SEGE	9.0 ± 4.5	6.0 (6.0–11.8)			
Grip strength (kg)	18.1 ± 6.3	16.9 (14.5–21.3)			
CS-30 (times)	4.2 ± 5.4	0 (0–9.0)			

Values are presented as mean ± standard deviation, median (25th–75th percentiles) or number (%).

n.s, not significant; LSA, life-space assessment; Hb-LSA, home-based LSA; FAI, Frenchay activities index; FIM, functional independence measure; SEADL, self-efficacy for activities of daily living; SEGE, self-efficacy scale on going out among community-dwelling elderly; CS-30, 30-second chair-stand test

The mean LSA score was 29.5, and the median LSA score was 27.0, indicating that most of the participants were not physically active outdoors once a week. The scores were higher in the Hb-LSA group than in the LSA group. Additionally, many participants engaged in more indoor life-space activities.

The FAI showed a high variability with a quartile range of 11.0 points, and many participants obtained a low median of 5.0 points.

Cognitive FIM had a mean score of 32.5, and a median of 34.0 points, indicating a high degree of independence; however, motor FIM had a mean of 72.3 points and a median of 76.0 points, indicating a degree of independence that was lower than that of cognitive FIM.

Table 2 indicates the correlation between Ikigai-9 scores and each assessment. There were significant positive correlations between Ikigai-9 scores and Hb-LSA, FAI, motor FIM, total FIM, SEADL, and SEGE scores. The correlation coefficient of SEADL with Ikigai-9 was the highest at 0.403, followed by FAI at 0.384, and motor FIM at 0.356. There was no correlation between the LSA of the physical activity index and grip strength or the CS-30 of the physical function assessment.

**Table 2.** Correlation between Ikigai and potential factors

	Age	LSA	Hb-LSA	FAI	Motor FIM	Cognitive FIM	Total FIM	SEADL	SEGE	Grip strength	CS-30
Ikigai-9											
*R*	0.060	0.174	0.339*	0.384*	0.356*	−0.011	0.339*	0.403*	0.307*	−0.101	0.216
*P* value	0.625	0.163	0.011	0.001	0.003	0.928	0.005	0.001	0.012	0.420	0.081

*P <0.05.

LSA, life-space assessment; Hb-LSA, home-based LSA; FAI, Frenchay activities index; FIM, functional independence measure; SEADL, self-efficacy for activities of daily living; SEGE, self-efficacy scale on going out among community-dwelling elderly; CS-30, 30-second chair-stand test

Table 3 reveals the multiple regression analysis with Ikigai-9 as the dependent variable; Hb-LSA, motor FIM, SEADL, and SEGE as independent variables; and sex as the adjusted variable. Preliminary analysis showed that the correlation coefficient between total FIM score and motor FIM was greater than 0.9. The motor-FIM, which showed a high correlation coefficient with the Ikigai-9, was used as the independent variable in this study.

**Table 3.** Multiple regression analysis results

Independent variable	Unstandardized regression coefficient (B)	Standardized partial regression coefficient (β)	95% CI	*P* value
FAI	0.400	0.422	0.184–0.615	<0.05

Multiple regression analysis, with adjusting for gender (male = 1, female = 0).

R^2^ = 0.255; adjusted R^2^ = 0.231.

FAI, Frenchay activities index; CI, Confidence interval

## Discussion

This study clarified the relationship between Ikigai and functioning and the factors associated with the influence of Ikigai among home-visit rehabilitation users aged 75 years and older who were receiving home medical and nursing care. This study revealed that the Ikigai-9 had a significant positive correlation with the Hb-LSA, FAI, motor FIM, total FIM, SEADL, and SEGE in home-visit rehabilitation users aged 75 years and older receiving medical and nursing care at home. In the multiple regression analysis in which Ikigai-9 was the dependent variable, FAI was selected. These results suggest that subjects with higher IADL may feel more Ikigai.

The results of the present study indicated that the mean Ikigai-9 score was lower than the mean of the Ikigai-9 score (27.3 ± 6.0) of a low-life purpose group of healthy older people^36)^.

In addition, the mean FAI score of the participants in this study was low (7.7 points). None of the participants exceeded the FAI cutoff value of 18 points^37)^.

Although the cognitive FIM score was good, the motor FIM score in our study was similar to that obtained in a previous study (72.8 points) conducted in older adults who had challenges in performing outdoor activities^38)^. The mean LSA of the analyzed participants was lower than the mean LSA of participants aged 75 years or older in a previous study^39)^, and none of the participants exceeded the cutoff value of 56 points for healthy older people^40)^. Many older adults require nursing care because their living spaces have decreased owing to a decline in ADL. Our study analysis revealed that the users of home-visit rehabilitation aged 75 years or older who had low ADL and IADL were primarily those with reduced physical activity.

Hb-LSA, not LSA, was the measure of physical activity most closely associated with Ikigai. In a previous study, Hb-LSA levels were lower in individuals who were not independent of indoor mobility than in those who were^26)^. The Hb-LSA may be more appropriate than the LSA for assessing physical activity in the living spaces of older people aged 75 years and older who require home medical and nursing care because few individuals are independent of indoor mobility and the Hb-LSA focuses on activities in the home. Multiple regression analysis suggested that, among the relevant items, the FAI, which assesses IADL, had an impact on Ikigai. The FAI assesses a variety of IADLs such as household roles, hobby activities, and employment.

Role and achievement motivation influence Ikigai^41)^. Additionally, hobbies and the presence or absence of a purpose in life may affect IADL^42)^. The IADL of older adults aged 75 years old changes rapidly^34)^. Hence, the FAI, which assesses IADL through multiple factors, may be selected for multiple regression analysis. In the present study, the association between the Ikigai and physical function was low. ADL was related to Ikigai; however, it was not selected as a factor. We discovered that even if physical function and ADL improved, Ikigai did not necessarily improve. For the subjects in the present study, the social factor Ikigai from social activities may have been more influential than the physical factor Ikigai from improved physical function. The details of Ikigai need to be further investigated and evaluated. Therefore, when conducting home-visit rehabilitation, not only physical function and ADL but also setting goals and practicing IADL are emphasized. Hence, the patient may perform the role and hobby activities that he or she desires which may lead to the improvement of Ikigai.

This study has 4 limitations. First, there were incomplete data for 54 subjects, which may have caused selection bias. Although this study was conducted by physical and occupational therapists with more than 4 years of experience, there may be many subjects who are easily fatigued or have difficulty communicating. In addition, the large number of assessment items and limited intervention time may have resulted in incomplete data. In the future, we plan to conduct a multicenter study with more participants by examining the selection of evaluation items and the conditions of participants and measures. Second, we were unable to compare the data by disease type. Similarly, we could not compare our results because of the small sample size. Users with intractable neurological incurable diseases and stroke have functional impairment and mental status that affect their QOL^43^^,^^44)^. In the future, we will consider the characteristics of each disease. Third, the adjusted coefficient of determination was low (0.231). Although IADL was extracted as a factor in the multiple regression analysis, other factors may have been related to Ikigai scores. Furthermore, owing to a lot of variation in the FAI, it is necessary to analyze the evaluation of IADL in-depth and investigate the factors that are indirectly related. In the future, we intend to collect more detailed data for further analysis. Fourth, this study may have evaluated only part of Ikigai. Although physical factors were not directly related to Ikigai in this study, it is possible that Ikigai was not fully assessed.

Further analysis is needed to evaluate Ikigai in more detail and to include indirect effects.

## Conclusion

Home-visit rehabilitation users aged 75 years or older who are receiving home medical and nursing care had lower values of Ikigai, ADL/IADL, and physical activity. IADL is related to Ikigai among home-visit rehabilitation users older than 75 years with low functioning. In-home-visit rehabilitation is necessary to improve IADL and Ikigai at home.

## Acknowledgments

The authors thank the staff at the Itabashi Rehabilitation Home Visit Nursing Station and the Nishinasuno General Home Care Center for their help with data collection.

## Funding

This work was supported by JSPS KAKENHI (grant no. 22K21121).

## Availability of data and materials

The data supporting the results of this study are available from the corresponding author upon reasonable request.

## Conflicts of Interest

The authors report no conflicts of interest.
